# Eddy covariance-based differences in net ecosystem productivity values and spatial patterns between naturally regenerating forests and planted forests in China

**DOI:** 10.1038/s41598-022-25025-4

**Published:** 2022-11-29

**Authors:** Xian-Jin Zhu, Ren-Xue Fan, Zhi Chen, Qiu-Feng Wang, Gui-Rui Yu

**Affiliations:** 1 College of Agronomy, Shenyang Agricultural University, Shenyang, 110866 China; 2 Synthesis Research Center of Chinese Ecosystem Research Network, Key Laboratory of Ecosystem Network Observation and Modeling, Institute of Geographic Sciences and Natural Resources Research, Chinese Academy of Sciences, 11A Datun Road, Chaoyang District, Beijing, 100101 China; 3 College of Resources and Environment, University of Chinese Academy of Sciences, Beijing, 100049 China; 4 Liaoning Panjin Wetland Ecosystem National Observation and Research Station, Shenyang, 110866 China

**Keywords:** Ecosystem ecology, Forest ecology

## Abstract

Net ecosystem productivity (NEP), the difference between gross primary productivity (GPP) and ecosystem respiration (ER), is the basis of forest carbon sinks. Revealing NEP differences between naturally regenerating forests (NF) and planted forests (PF) can benefit for making carbon neutrality strategies. Based on 35 eddy covariance measurements in China, we analyzed NEP differences in values and spatial patterns between NF and PF. The results showed that NF had slightly lower NEP than PF, resulting from the high stand age (SA) and soil fertilizer, while their differences were not significant (p > 0.05). The increasing latitude decreased mean annual air temperature thus decreased GPP both in NF and PF. However, the higher SA and soil fertilizer in NF made most GPP release as ER thus induced no significant NEP spatial variation, while lower SA and soil fertilizer in PF made NEP spatially couple with GPP thus showed a decreasing latitudinal pattern. Therefore, stand characteristics determined the differences in NEP values but indirectly affected the differences in NEP spatial variations through altering GPP allocation. The decreasing latitudinal pattern of NEP in PF indicates a higher sequestration capacity in the PF of South China. Our results provide a basis for improving the forest carbon sequestration.

## Introduction

Forests have the strongest carbon sequestrating capacity of all land covers^1–3^, playing an important role in mitigating the atmosphere CO_2_ increasing rate^4–7^. Forest net ecosystem productivity (NEP), the difference between gross primary productivity (GPP) and ecosystem respiration (ER), is the basis of forest carbon sinks^8–10^. Analyzing the values of forest NEP can address the roles of forests in sequestering CO_2_, while analyzing the spatial patterns of forest NEP can reveal the differences in forest carbon sequestration among regions. Both actions benefit forest carbon management, which aids carbon neutrality^11,12^ thus climate change mitigation^13–15^.

Many works have investigated NEP values and spatial patterns of forests through eddy covariance measurements^16–24^, which can be performed in-situ and continuously measure NEP without damaging forest sites. Forests are found to have a positive NEP in most regions^7,25,26^, and even old forests sequester CO_2_ from the atmosphere^27,28^. In addition, climatic variables, including the mean annual air temperature (MAT) and mean annual precipitation (MAP), are found to influence forest NEP spatial patterns^17,19,20,24^, which are also affected by nitrogen deposition^29,30^, forest age^31–33^, fire^34^, etc. Forests can be categorized into naturally regenerating forests (NF) and planted forests (PF) based on their origins^35^. Revealing the differences between NF and PF can improve our understanding of the roles of different forest types in mitigating climate change. However, little attention has been paid to the differences in NEP values and spatial patterns between NF and PF, inhibiting our understanding of the roles of different forest types in mitigating climate change.

China has a large forest area with forest coverage of 22.96%^36^. In addition to protecting existing NF^37^, China invests great efforts in planting trees, thus creating a great deal of PF^1,38^. Therefore, NF and PF are widely distributed throughout Chinese terrestrial ecosystems^36^, which provides an ideal platform to analyze their differences in NEP values and spatial patterns. Chinese Terrestrial Ecosystem Flux Research Network (ChinaFLUX) has promoted eddy covariance measurements in China^39,40^. Many eddy covariance measurements have been conducted in NF and PF, enabling the analysis of NEP differences between NF and PF. In addition, constrained by the Qinghai-Tibet Plateau uplift and Asian monsoon, China has a unique climate gradient^41^. Analyzing the NEP differences between NF and PF in China can improve our understanding of global forest carbon sequestration capacity.

Therefore, after integrating eddy covariance measurements in Chinese forests, we analyzed the differences in NEP values and spatial patterns between NF and PF. The main objectives were to clarify (1) the differences in carbon sequestration capacities between NF and PF and (2) the differences in spatial patterns in carbon sequestration capacity between NF and PF. Our results can be beneficial for understanding the roles of forests in sequestering CO_2_, thereby aiding carbon management strategies aiming at increasing forest carbon uptake.

## Results

### Differences in environmental factors

**Figure 1:**
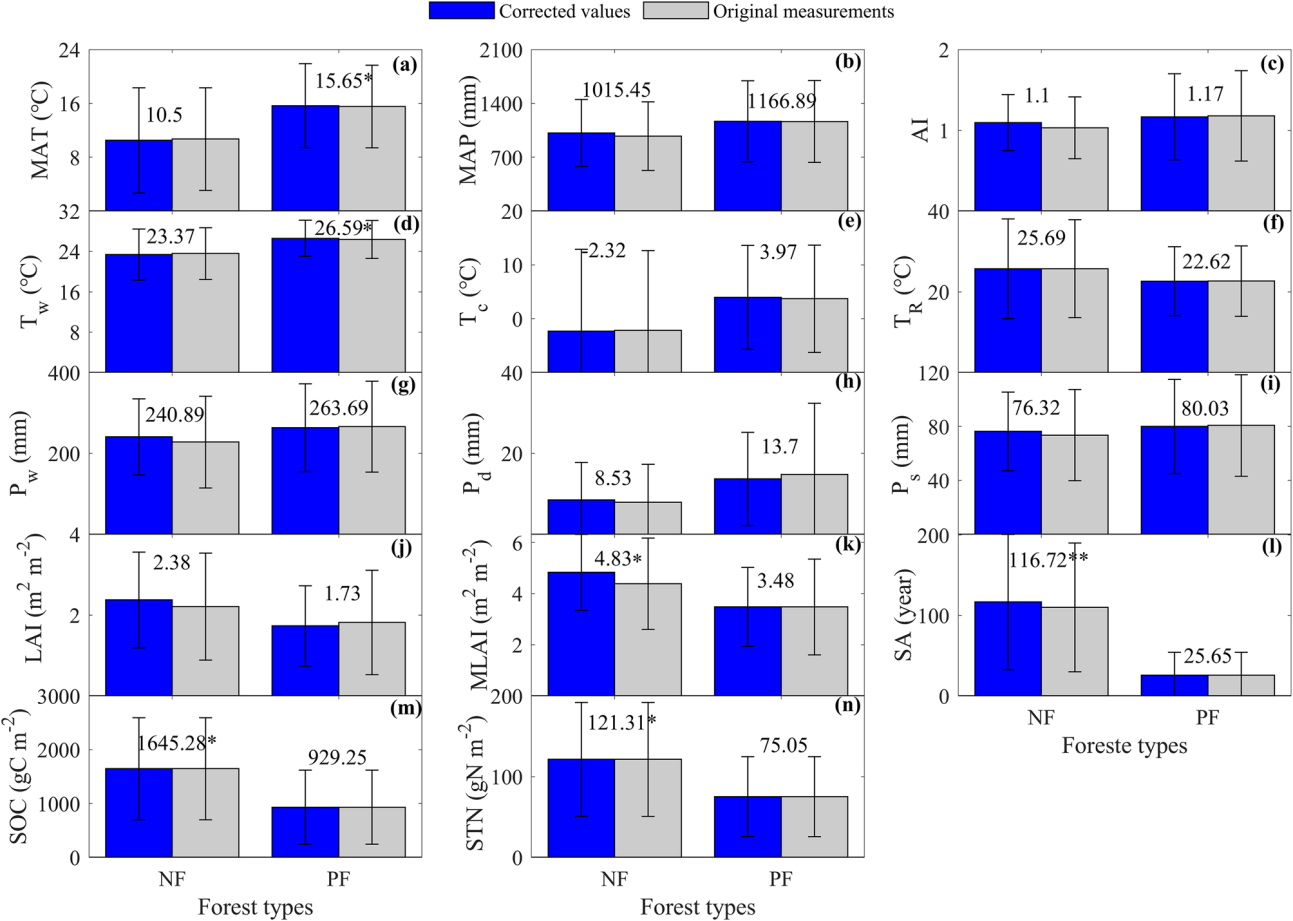
The differences in environmental factors between naturally regenerating forests (NF) and planted forests (PF) in China. The environmental factors include three annual climatic factors (**a**–**c**), three seasonal temperature factors (**d**–**f**), three seasonal precipitation factors (**g**–**i**), three biotic factors (**j**–**l**), and two soil factors (**m**,**n**). Three annual climatic factors include mean annual air temperature (MAT, **a**), mean annual precipitation (MAP, **b**), and aridity index (AI, **c**) defined as the ratio of MAP to annual potential evapotranspiration. Three seasonal temperature factors include the temperature of the warmest month (T_w_, **d**), the temperature of the coldest month (T_c_, **e**), temperature annual range (T_R_, **f**). Three seasonal precipitation factors include precipitation of the wettest month (P_w_, **g**), precipitation of the driest month (P_d_, h), and precipitation seasonality (Ps, **i**) defined as the standard deviation of monthly precipitation during the measuring year. Three biological factors include the mean annual leaf area index (LAI, **j**), the maximum leaf area index (MLAI, **k**), and stand age (SA, **l**). Two soil factors include soil organic carbon content (SOC, **m**) and soil total nitrogen content (STN, **n**). The differences are tested for each variable with one-way analysis of variance (ANOVA), where * and ** indicate significant differences between forest types at significance levels of *α* = 0.05 and *α* = 0.01, respectively. The corrected values are mean values during 2003–2019 after correcting the original measurements with the interannual trend (See methods), which are listed in each panel, while original measurements are mean values during the measuring period of each ecosystem, which are not shown in each panel.

Environmental factors showed value differences between forest types, while the significance of differences differed among variables, which were both found with corrected values and original measurements (Fig. 1).

For annual climatic factors, the significant difference between NF and PF only appeared in MAT (Fig. 1a). The mean MAT of NF was 10.50 ± 7.81 °C, which was significantly lower than that of PF (15.65 ± 6.23 °C) (p < 0.05). However, the MAP and aridity index (AI, defined as the ratio of MAP to annual potential evapotranspiration) showed no significant difference between forest types (Fig. 1b,c), although their values differed.

For seasonal climatic factors, only the temperature of the warmest month (T_w_) showed a significant difference between NF and PF (p < 0.05) (Fig. 1d), while other factors, including the seasonal temperature factors and the seasonal precipitation factors, showed no significant differences between NF and PF (Fig. 1e–i).

However, the differences in biological factors showed divergent significance among factors (Fig. 1j–l). Mean annual leaf area index (LAI) showed no significant difference between NF and PF (Fig. 1j), while the maximum leaf area index (MLAI) and stand age (SA) significantly differed between forest types (Fig. 1k–l). The MLAI of NF (4.83 ± 1.49 m^2^ m^−2^) was significantly higher than that of PF (p < 0.05) (Fig. 1k). NF also had a significantly higher SA (116.72 ± 84.14 years) than PF (p < 0.01), which was 25.65 ± 28.48 years (Fig. 1l).

Soil factors showed significant differences between forest types, with a higher content appearing in NF (Fig. 1m,n). The soil organic carbon content (SOC) of NF was 1645.28 ± 949.18 gC m^−2^, which was significantly higher (p < 0.05) than that of PF (929.25 ± 686.67 gC m^−2^). The soil total nitrogen content (STN) of NF, which was 121.31 ± 70.60 gN m^−2^, was also significantly higher (p < 0.05) than that of PF (75.05 ± 49.49 gN m^−2^).

### Differences in NEP values

**Figure 2:**
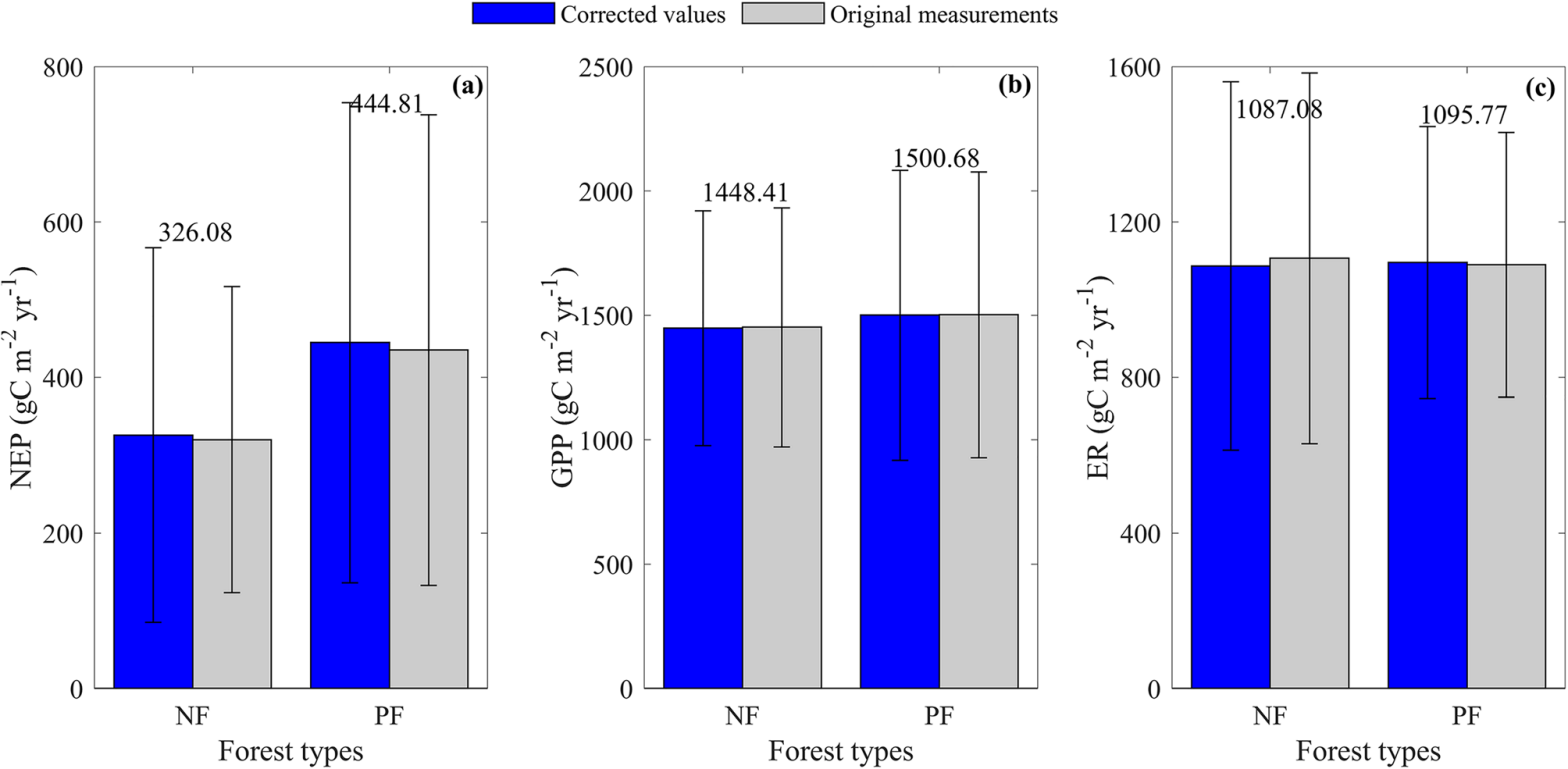
The differences in carbon flux values between naturally regenerating forests (NF) and planted forests (PF) in China. The carbon fluxes include net ecosystem productivity (NEP, **a**), gross primary productivity (GPP, **b**), and ecosystem respiration (ER, **c**). The differences are tested for each flux with one-way analysis of variance (ANOVA) and analysis of covariance (ANCOVA) by fixing other variables as a covariant. All results show no significant difference between NF and PF (p > 0.05). The corrected values and original measurements are same to those in Fig. 1.

Carbon flux values differed between forest types but their differences were not statistically significant, which were both true with corrected values and original measurements (Fig. 2).

The mean NEP of NF was 326.08 ± 240.87 gC m^−2^ year^−1^, which was lower than that of PF (444.81 ± 308.76 gC m^−2^ year^−1^), while the results from one-way analysis of variance (ANOVA) indicated that NEP values did not significantly differ between forest types (F = 1.6, p > 0.05) (Fig. 2a). Even considering the significant effects of MAT on NEP, the results from analysis of covariance (ANCOVA) by fixing MAT as a covariant also suggested that no significant difference was found in NEP values between forest types (F = 2.09, p > 0.05). Fixing other variables as a covariant also found no significant differences in NEP values between NF and PF.

The mean GPP value of NF was 1448.41 ± 471.13 gC m^−2^ year^−1^, which was slightly lower than that of PF (1500.68 ± 582.84 gC m^−2^ year^−1^) (Fig. 2b), while ANOVA results indicated that the GPP value of NF showed no significant difference from that of PF (F = 0.07, p > 0.05) (Fig. 2b). Even considering the significant effects of MAT on GPP, ANCOVA results obtained by fixing MAT as a covariant also suggested that GPP values did not significantly differ between forest types (F = 1.52, p > 0.05). Fixing other variables as a covariant also drew a similar result.

The mean ER value of NF (1087.08 ± 473.42 gC m^−2^ year^−1^) was slightly lower than that of PF (1095.77 ± 349.83 gC m^−2^ year^−1^). ANOVA results also indicated that ER values did not significantly differ between forest types (F = 0.00, p > 0.05) (Fig. 2c). Even considering the significant effects of MAT on ER, ANCOVA results obtained by fixing MAT as a covariant also suggested that ER values did not significantly differ between forest types (F = 0.01, p > 0.05). Fixing other variables as a covariant also drew a similar result.

Therefore, NF showed a lower NEP resulting from the lower GPP than PF, while their differences were not statistically significant (Fig. 2).

### Differences in NEP latitudinal patterns

**Figure 3:**
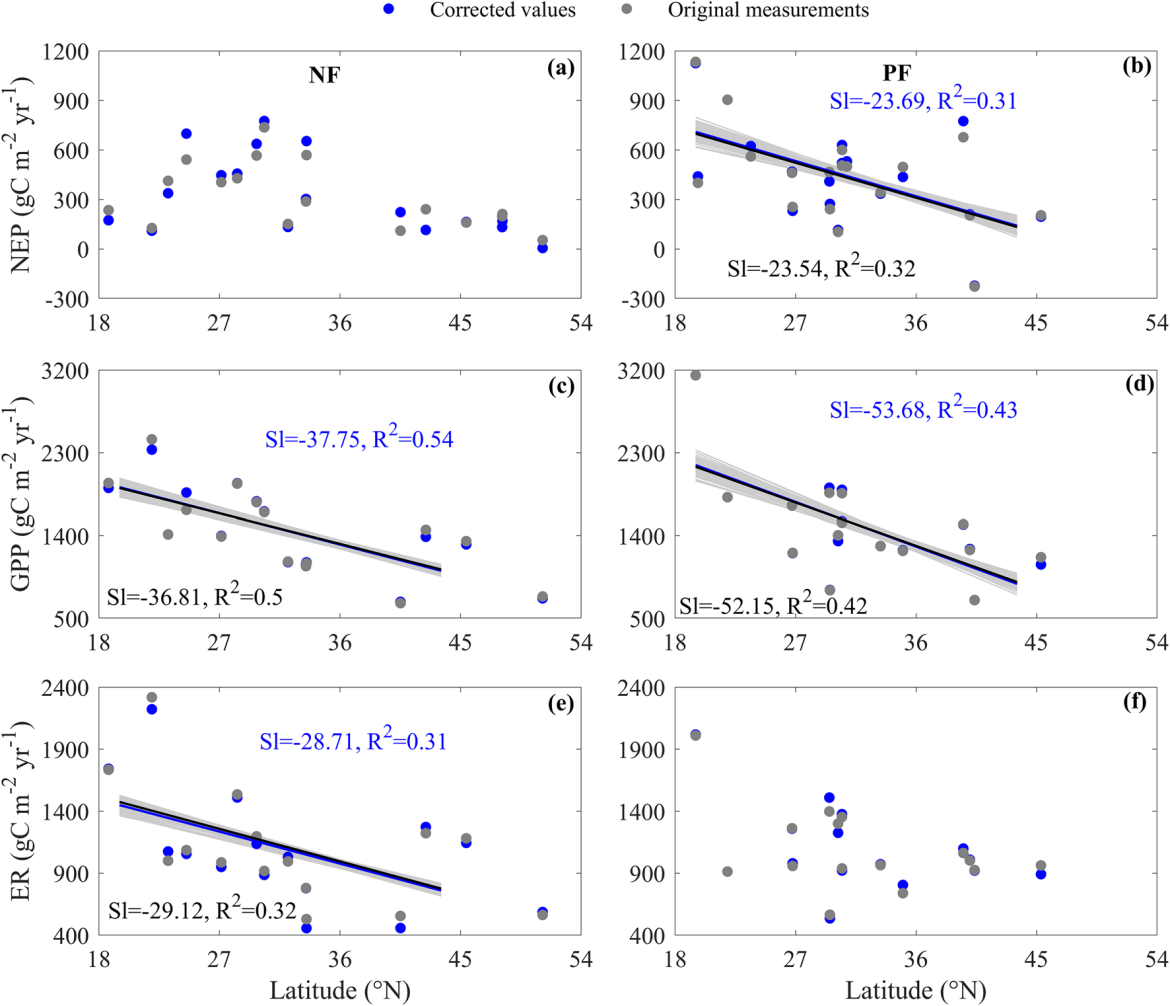
The latitudinal patterns of carbon fluxes over Chinese naturally regenerating forests (NF) and planted forests (PF). The carbon fluxes include net ecosystem productivity (NEP, **a**,**b**), gross primary productivity (GPP, **c**,**d**), and ecosystem respiration (ER, **e**,**f**). Each panel is drawn with the corrected values (blue points) and original measurements (grey points), respectively. The blue and black lines represent the regression lines calculated from the corrected values and original measurements, respectively, with their regression statistics listed in blue and black letters. Only the regression slope (Sl) and R^2^ of each regression are listed. The grey lines represent the regressions between carbon fluxes added by random errors and latitude. Only significant (p < 0.05) regression lines are drew in each panel.

Carbon fluxes showed divergent latitudinal patterns between NF and PF, while their latitudinal patterns varied among carbon fluxes, which were both found with corrected values and original measurements (Fig. 3).

NEP showed no significant latitudinal pattern among NFs (Fig. 3a), while that of PF exhibited a significant decreasing latitudinal pattern (Fig. 3b). With increasing latitude, the NEP of NF showed no significant spatial variation. However, the increasing latitude caused the NEP of PF to significantly decrease. Each unit increase in latitude led to a 23.69 gC m^−2^ year^−1^ decrease in NEP, with an R^2^ of 0.31.

The GPP of NF and PF both exhibited significant decreasing latitudinal patterns with similar decreasing rates (Fig. 3c,d). With increasing latitude, the GPP of NF significantly decreased. Each unit increase in latitude led to a 37.75 gC m^−2^ year^−1^ decrease in GPP, with an R^2^ of 0.54. The increasing latitude significantly decreased the GPP of PF. Each unit increase in latitude decreased the GPP of PF at a rate of 53.68 gC m^−2^ year^−1^, with an R^2^ of 0.43. Although the decreasing rates differed in values between NF and PF, their differences were not statistically significant (F = 0.71, p > 0.05).

The ER of NF showed a significant decreasing latitudinal pattern (Fig. 3e), while that of PF exhibited no significant latitudinal pattern (Fig. 3f). The increasing latitude caused the ER of NF to significantly decrease. Each unit increase in latitude led to a 28.71 gC m^−2^ year^−1^ decrease in ER, with an R^2^ of 0.31. However, the increasing latitude contributed little to the ER spatial variation of PF (p > 0.05).

In addition, the latitudinal patterns of carbon fluxes and their differences between forest types were also obtained with the original measurements (Fig. 3, grey points). The latitudinal patterns of random error adding carbon fluxes were comparable to those of our corrected carbon fluxes (Fig. 3), which confirmed that the latitudinal patterns of carbon fluxes and their differences between forest types would not be affected by the uncertainties in generating the corrected carbon fluxes.

Therefore, among NFs, the similar decreasing latitudinal patterns of GPP and ER meant that NEP showed no significant latitudinal pattern, while the significant decreasing latitudinal pattern of GPP and no significant latitudinal pattern of ER caused NEP to show a decreasing latitudinal pattern among PFs.

### Differences in the environmental effects on NEP spatial variations

Environmental factors, including the annual climatic factors, seasonal temperature factors, seasonal precipitation factors, biological factors, and soil factors, exerted divergent effects on the spatial variations of NEP and its components, which also differed between forest types (Table 1). No factor was found to affect that the spatial variation of NEP among NFs, while most annual and seasonal climatic factors were found to affect that among PFs. The spatial variations of GPP and ER among NFs were both affected by most annual and seasonal climatic factors and LAI, while those among PFs were primarily shaped by most annual and seasonal climatic factors. Though LAI showed no significant effect on GPP and ER spatial variations among PFs, SA exerted a significant negative effect. In addition, the spatial variations of soil variables contributed little to the spatial variations of carbon fluxes. Therefore, among NFs, most annual and seasonal climatic factors and LAI were found to affect GPP and ER spatial variations, while no factor was found to significantly influent the NEP spatial variation. However, among PFs, most annual and seasonal climatic factors were found to affect the spatial variations of NEP and its components, while LAI showed no significant effect. Using the original measurements also generated the similar correlation coefficients (Supplementary Table S1).

**Table 1:** Correlation coefficients between carbon fluxes and environmental factors in naturally regenerating forests (NF) and planted forests (PF).

Environmental factors	NEP		GPP		ER	
	NF	PF	NF	PF	NF	PF
MAT	0.31	*0.56*	**0.70**	**0.75**	*0.62*	**0.69**
MAP	0.30	*0.52*	**0.70**	**0.72**	*0.61*	*0.57*
AI	0.28	0.38	*0.61*	*0.54*	0.50	0.42
Tw	0.36	0.44	0.45	**0.66**	0.33	**0.72**
Tc	0.48	**0.66**	**0.72**	**0.74**	*0.53*	*0.52*
Tr	− 0.44	− *0.56*	**− 0.68**	*− 0.53*	− 0.50	− 0.25
Pw	0.08	*0.51*	*0.55*	**0.82**	*0.56*	**0.70**
Pc	0.43	0.20	0.40	0.45	0.23	*0.52*
Ps	0.02	*0.53*	*0.55*	**0.82**	*0.58*	**0.65**
LAI	− 0.03	0.44	**0.66**	0.50	**0.73**	0.32
MLAI	− 0.06	0.23	0.19	0.26	0.33	0.15
SA	0.15	− 0.03	0.30	− 0.35	0.16	− *0.54*
SOC	0.30	− 0.29	− 0.02	− 0.36	− 0.15	− 0.24
STN	0.30	− 0.39	− 0.09	− 0.29	− 0.21	− 0.11

Carbon fluxes include net ecosystem productivity (NEP), gross primary productivity (GPP), and ecosystem respiration (ER). The abbreviations of the environmental factors are the same as those in the legend of Fig. 1. Italics and bold indicate significant correlation coefficients at *α* = 0.05 and *α* = 0.01, respectively.

Given the high correlations among annual climatic factors and seasonal climatic factors (Supplementary Table S2), the partial correlation analysis was applied to determine which factors should be employed to reveal the mechanisms underlying the spatial variations of NEP. Partial correlation analysis showed that MAT and MAP exerted the most important roles in spatial variations of NEP and its components (Table 2). After controlling MAT (or MAP), other factors seldom showed significant correlation with carbon fluxes, especially fixing MAT (Table 2). In addition, MAT and MAP exerted similar effects on the spatial variations of NEP and its components (Table 1). Using the original measurements also generated the similar partial correlation coefficients (Supplementary Table S3). Therefore, we only presented the effects of MAT on carbon flux spatial variations and their differences between forest types in detail.

**Table 2:** Partial correlation coefficients between carbon fluxes and environmental factors in naturally regenerating forests (NF) and planted forests (PF) with fixing mean annual air temperature (MAT) or mean annual precipitation (MAP).

Fixed variable	Environmental factors	NEP		GPP		ER	
		NF	PF	NF	PF	NF	PF
MAT	MAP	0.07	0.24	0.29	0.40	0.20	0.17
	AI	0.12	0.13	0.28	0.23	0.15	0.09
	Tw	0.19	0.00	− 0.22	0.13	− 0.32	0.41
	Tc	0.53	0.42	0.27	0.33	− 0.12	− 0.06
	Tr	− 0.34	− 0.34	− 0.31	− 0.20	− 0.05	0.19
	Pw	− 0.21	0.21	0.11	*0.61*	0.22	0.43
	Pc	0.32	− 0.09	0.09	0.13	− 0.11	0.29
	Ps	− 0.27	0.29	0.16	**0.67**	0.29	0.39
	LAI	− 0.34	0.32	0.38	0.28	0.55	0.04
	MLAI	− 0.15	0.25	0.22	0.26	0.37	0.09
	SA	0.13	0.32	0.33	0.24	0.14	− 0.21
	SOC	0.39	− 0.04	0.44	0.15	0.17	0.24
	STN	0.37	− 0.12	0.29	0.21	0.06	0.39
MAP	MAT	0.11	0.34	0.29	0.49	0.26	0.45
	AI	0.02	− *0.60*	− 0.16	− **0.69**	− 0.24	− 0.47
	Tw	0.21	0.27	− 0.08	0.51	− 0.16	*0.62*
	Tc	0.42	*0.49*	0.35	0.34	0.08	0.09
	Tr	− 0.34	− 0.29	− 0.39	0.02	− 0.15	0.30
	Pw	− 0.36	0.07	− 0.12	*0.56*	0.11	*0.56*
	Pc	0.32	− 0.14	− 0.09	− 0.15	− 0.26	0.19
	Ps	− 0.37	0.16	0.01	*0.56*	0.22	0.39
	LAI	− 0.31	0.17	0.38	0.14	*0.56*	− 0.01
	MLAI	− 0.17	0.10	0.06	0.15	0.25	0.03
	SA	0.12	− 0.14	0.28	− 0.37	0.10	− *0.57*
	SOC	0.39	− 0.45	0.34	− 0.39	0.09	− 0.20
	STN	0.40	− 0.44	0.32	− 0.24	0.07	− 0.01

Carbon fluxes include net ecosystem productivity (NEP), gross primary productivity (GPP), and ecosystem respiration (ER). The abbreviations of the environmental factors are the same as those in the legend of Fig. 1. Italics and bold indicate significant correlation coefficients at *α* = 0.05 and *α* = 0.01, respectively.

**Figure 4:**
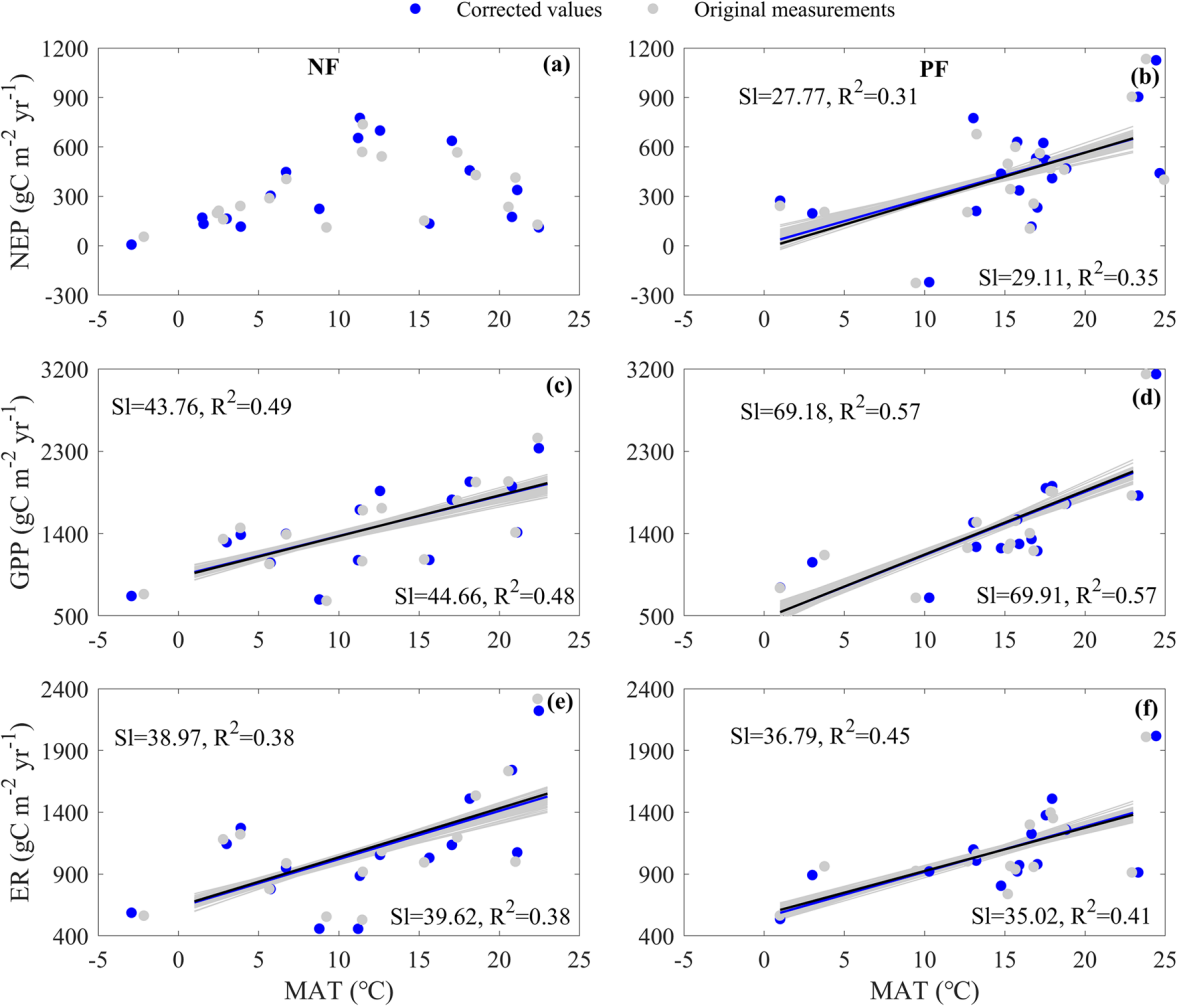
The effects of mean annual air temperature (MAT) on the spatial variations of carbon fluxes over Chinese naturally regenerating forests (NF) and planted forests (PF). The carbon fluxes include net ecosystem productivity (NEP, **a**,**b**), gross primary productivity (GPP, **c**,**d**), and ecosystem respiration (ER, **e**,**f**). Each panel is drawn with the corrected values (blue points) and original measurements (grey points), respectively. The blue and black lines represent the regression lines calculated from the corrected values and original measurements, respectively, with their regression statistics listed in blue and black letters. Only the regression slope (Sl) and R^2^ of each regression are listed. The grey lines represent the regressions between carbon fluxes added by random errors and latitude. Only significant (p < 0.05) regression lines are drew in each panel.

The increasing MAT increased carbon fluxes, while the increasing rates differed between forest types (Fig. 4). The increasing MAT contributed little to the NEP spatial variation of NF but raised the NEP of PF (Fig. 4a,b). Each unit increase in MAT caused the NEP of PF to increase at a rate of 27.77 gC m^−2^ year^−1^, with an R^2^ of 0.31 (Fig. 4b). The increasing MAT significantly raised GPP in NF and PF (Fig. 4c,d). For NF, each unit increase in MAT increased GPP at a rate of 43.76 gC m^−2^ year^−1^, with an R^2^ of 0.49 (Fig. 4c), while each unit increase in MAT increased the GPP of PF at a rate of 69.18 gC m^−2^ year^−1^, with an R^2^ of 0.57 (Fig. 4d). The GPP increasing rates did not significantly differ between NF and PF (F = 1.52, p > 0.05). The increasing MAT also raised ER in both NF and PF (Fig. 4e,f), whose increasing rates were 38.97 gC m^−2^ year^−1^ (Fig. 4e) and 36.79 gC m^−2^ year^−1^ (Fig. 4f), respectively, while their differences were not statistically significant (F = 0.01, p > 0.05). In addition, using the original measurements also generated the similar spatial variations and their differences between forest types (Fig. 4). Furthermore, the random error adding carbon fluxes responded similarly to those of our correcting carbon fluxes (Fig. 4), indicating that the effects of MAT on carbon fluxes would not be affected by the uncertainties in our correcting carbon fluxes. Therefore, the similar responses of GPP and ER to MAT made MAT contribute little to NEP spatial variations among NFs, while GPP and ER showed divergent response rates to MAT, which made NEP increase with MAT among PFs.

### Differences in the spatial couplings between carbon fluxes

**Figure 5:**
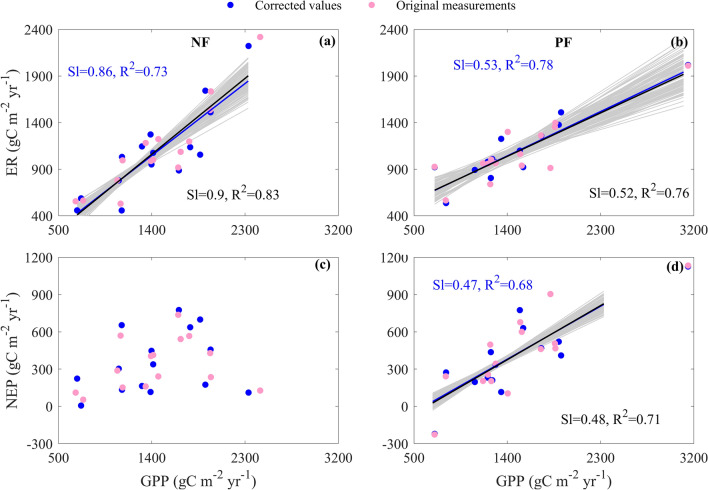
The spatial couplings between carbon fluxes over Chinese naturally regenerating forests (NF) and planted forests (PF). The spatial couplings include the coupling between gross primary productivity (GPP) and ecosystem respiration (ER) (**a**,**b**) and that between GPP and net ecosystem productivity (NEP) (**c**,**d**). Each panel is drawn with the corrected values (blue points) and original measurements (pink points), respectively. The blue and black lines represent the regression lines calculated from the corrected values and original measurements, respectively, with their regression statistics listed in blue and black letters. Only the regression slope (Sl) and R^2^ of each regression are listed. The grey lines represent the regressions between carbon fluxes added by random errors and latitude. Only significant (p < 0.05) regression lines are drew in each panel.

The spatial couplings between carbon fluxes differed between NF and PF (Fig. 5).

GPP and ER were spatially coupled in both NF and PF, while the responses of ER to GPP differed between NF and PF (Fig. 5a,b). With increasing GPP, the ER of NF linearly increased at a rate of 0.86 gC m^−2^ year^−1^, with an R^2^ of 0.73 (Fig. 5a). The increasing GPP also caused the ER of NF to linearly increase, with an increasing rate of 0.53 gC m^−2^ year^−1^ and an R^2^ of 0.78 (Fig. 5b). The ER increasing rate of NF was significantly higher than that of PF (F = 5.78, p < 0.05).

However, GPP was only spatially coupled with NEP in PF (Fig. 5c,d). The increasing GPP contributed little to the NEP spatial variation of NF, whereas the increasing GPP caused NEP to linearly increase at a rate of 0.47 gC m^−2^ year^−1^ in PF, with an R^2^ of 0.68 (Fig. 5d).

In addition, the spatial couplings of carbon fluxes and their differences between forest types were also obtained with the original measurements (Fig. 5, pink symbols) and the error adding carbon fluxes (Fig. 5).

### Comprehensive understanding on differences in NEP spatial patterns

Based on NEP latitudinal patterns, effects of environmental factors on NEP spatial variations, and the couplings between carbon fluxes, we found that NEP spatial patterns sourced from the spatial patterns of GPP and ER, which resulted from the increasing MAT induced by the decreasing latitude (Figs. 3, 4, 5).

The increasing latitude induced a decreasing MAT (Supplementary Table S2), which showed no significant difference between NF and PF. With increasing latitude, MAT showed a significant decreasing trend at a rate of 0.70 °C, with an R^2^ of 0.68 (Eq. (1)).

1 $${\text{MAT}} = 35.77 - 0.70\;{\text{Latitude}},\;{\text{R}}^{2} = 0.68,\;{\text{n}} = 35,\;{\text{p}} < 0.01$$

The increasing MAT exerted a positive effect on the spatial variation of GPP (Fig. 4), which also showed no significant difference between NF and PF. The increasing MAT raised GPP at a rate of 52.37 gC m^−2^ year^−1^, with an R^2^ of 0.48 (Eq. (2)).

2 $${\text{GPP}} = 779.6 + 52.37\;{\text{MAT}},\;{\text{R}}^{2} = 0.48,\;{\text{n}} = 30,\;{\text{p}} < 0.01$$

The increasing GPP exerted a positive effect on ER spatial variation, while its effects significantly differed between NF and PF (Fig. 5). ER spatial variation was jointly shaped by the forest type, GPP, and their interaction, with an R^2^ of 0.72 (Eq. (3)). NF showed a lower intercept and a higher increasing rate, while PF had a higher intercept and a lower increasing rate.

3 $${\text{ER}} = 302.03 - 460.31\;{\text{Forest}} + 0.53\;{\text{GPP}} + 0.33\;{\text{Forest}} \times {\text{GPP}},\;{\text{R}}^{2} = 0.72,\;{\text{n}} = 30,\;{\text{p}} < 0.01$$

where Forest represents the forest type with NF of 1 and PF of 0, while Forest × GPP reflects the interaction between forest type and GPP, which is set to GPP at NF and 0 at PF.

With the joint effects of GPP and ER, NEP spatial variation was also primarily shaped by the spatial variation of GPP, while the effects of GPP significantly differed between NF and PF (Fig. 5). NEP spatial variation was jointly shaped by the forest type, GPP, and their interaction, with an R^2^ of 0.42 (Eq. (4)).

4 $${\text{NEP}} = - 283.42 + 417.52{\text{Forest}} + 0.47{\text{GPP}} - 0.33{\text{Forest}} \times {\text{GPP}},\;{\text{R}}^{2} = 0.42,\;{\text{n}} = 30,\;{\text{p}} < 0.01$$

Using the original measurements, we also got the similar equations (Supplementary Text S1).

Therefore, the increasing latitude decreased MAT thus GPP, which did not significantly differ between NF and PF. However, the differences in SA and soil variables made the portion of GPP allocated to ER thus NEP significantly differ, which made NEP show divergent spatial patterns between NF and PF.

## Discussion

In this study, we found a lower but stable NEP in NF, while a higher but variable NEP was found in PF (Fig. 2). The differences in NEP resulted from the higher GPP and comparable ER in PF, which could be attributed to 4 aspects. First, plant compositions differed between NF and PF, thus introducing divergent GPPs. As naturally regenerating ecosystems, NF were primarily composed of indigenous plants, which adapted to the local climate. Therefore, NF showed a mild carbon sequestration capacity indicated by a lower GPP. However, PF were dominated by planted fast-growing trees such as Chinese fir^42^, poplar^43^ and rubber^44^, which are characterized by fast growth and thus a higher GPP. Second, the leaf type may contribute to the differences in GPP. NF were evenly composed of evergreen (9/17) and deciduous (8/17) forests, while PF were primarily composed of evergreen forests (12/18), causing PF to have a higher GPP with a longer carbon sequestration period. Third, SA differed between NF and PF, introducing different substrate amounts and thus ERs. NF generally had a higher SA, thus introducing a higher SOC (Fig. 1), which caused NF to have a higher ER. Fourth, a higher MAT in PF smoothed the differences in ER between NF and PF. PF experienced a significantly higher MAT than NF, which meant that higher ER thus smoothed the differences between NF and PF.

Although NEP values differed between NF and PF, their differences were not statistically significant (Fig. 2a), which could be attributed to the large range of NEP values as the ecosystems distributed in a wide area and the number of forest observations was limited. The ecosystems used in this study widely distributed from tropical seasonal rainforests to cool temperate evergreen needleleaf forests, with MATs ranging from -4 to 25 °C, which made NEP values vary substantially. For example, the NEP of NF ranged from 6.5 to 775.58 gC m^−2^ year^−1^ (Supplementary Table S4), while that of PF floated from − 221.61 to 1125.6 gC m^−2^ year^−1^. Although we made our best to collect eddy covariance measurements in China, only 35 forest measurements were gathered. The limited observations may have introduced biases in estimating the statistics of the total and thus may contribute to the lack of significant differences in NEP between forest types. Further studies should gather more observations to validate the differences in NEP values between forest types. However, our results could conservatively confirm that NF and PF showed comparable carbon sequestration capacities.

In addition to differences in NEP values, we found that the NEP of NF responded little to environmental factors and thus showed no significant latitudinal pattern, while the NEP of PF significantly decreased with increasing environmental factors and thus showed a decreasing latitudinal pattern. The divergent spatial patterns of NEP between NF and PF resulted from the divergent portions of GPP allocating to ER, which induced the divergent ER responses characterized by different forest types. The increasing latitude decreased MAT in spatial thus reduced GPP, which was both found in NF and PF. However, the differences in stand characteristics like SA and soil variables made GPP allocation differed between NF and PF. In NF, carbon sequestration (GPP) and carbon release (ER) were in a dynamic equilibrium after a long period of coevolution. ER and GPP thus responded similarly to environmental factors, introducing the spatially coupling between GPP and ER (Fig. 5a). The comparable responses between GPP and ER resulted in a relatively stable difference as NEP and thus no significant latitudinal pattern (Fig. 3a). While for PF, most portion of GPP was accumulated as PF were in an active growing stage with the fast-growing trees. In addition, the lower SOC in PF resulted in a small portion of heterotrophic respiration. Besides the spatial coupling between GPP and ER, GPP and NEP thus also spatially coupled (Fig. 5d). The NEP of PF increased with environmental factors and thus showed a decreasing latitudinal pattern (Fig. 3b).

Therefore, the stand characteristics like stand age (SA) or soil variables directly determined the difference in NEP values between NF and PF, while they seldom directly affected NEP spatial variations. However, the stand characteristics inducing GPP allocation differed between forest types, which resulted in the divergent spatial patterns of NEP.

The latitudinal patterns of NEP suggest that the carbon sequestration capacities of NF did not vary among regions while those of PF decreased with increasing latitude. Therefore, the PF of South China had a higher carbon sequestration capacity, which indicated that they may play an important role in carbon neutrality and climate change mitigation. However, we only analyzed the eddy covariance measurements, while other approaches, such as resource inventory, were employed to quantify the carbon sink values^45,46^. Further analysis should combine different approaches to further validate our results.

## Conclusions

Based on eddy covariance measurements from 35 forests in East China, we analyzed the differences in net ecosystem productivity (NEP) values and spatial patterns between naturally regenerating forests (NF) and planted forests (PF). The results showed that stand characteristics determined the differences in NEP values but indirectly affected the differences in NEP spatial patterns through influencing the allocation of gross primary productivity (GPP) to NEP. NF showed slightly lower NEP than PF, while their differences were not statistically significant. NEP of NF showed no significant spatial patterns as most GPP was released through ecosystem respiration (ER), while NEP of PF showed a significant decreasing latitudinal pattern as the close coupling between GPP and NEP. Therefore, NF and PF had comparable carbon sequestration capacities, while their spatial patterns obviously differed. The carbon sequestration capacities of NF did not vary among regions while those of PF decreased with increasing latitude, indicating that the PF of South China had a higher carbon sequestration capacity. Our results provide a basis for improving the carbon sequestration capacity of forests.

## Methods

### Site information

**Figure 6:**
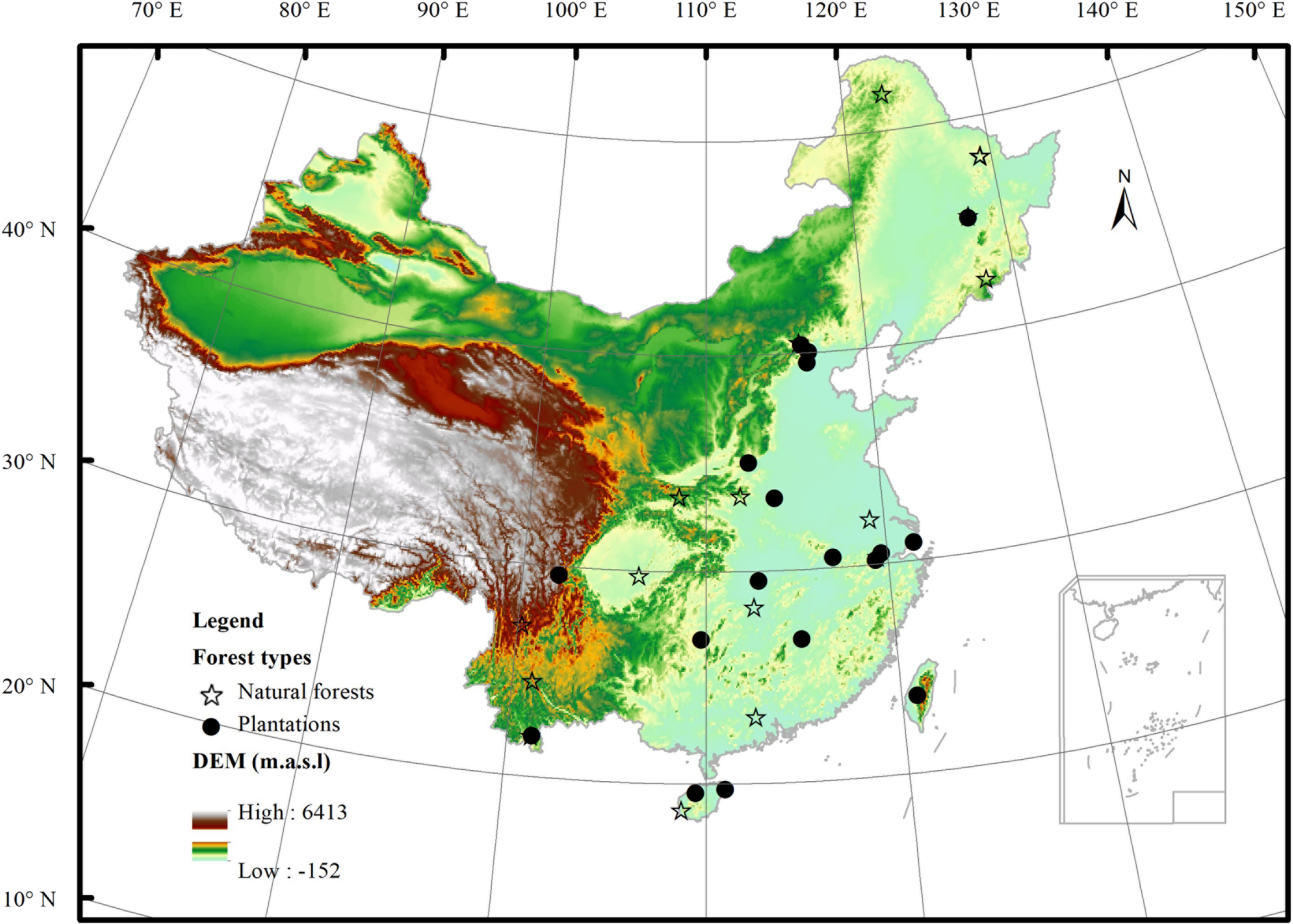
The spatial distribution of ecosystems used in this study. The maps are made by ArcGIS 10.0 software (http://www.esri.com/software/arcgis).

Using “eddy covariance” and “forest” as keywords, we searched the published works from www.cnki.net and www.isiknowledge.com and selected all studies that measured NEP and its components. All searched studies were screened, and only results containing more than 1 year of eddy covariance measurements (including 1 year) were extracted. The NEP and its components were extracted to build a comprehensive carbon flux dataset, which contained 35 ecosystems. The forests were categorized into NF and PF following FAO criteria^35^, where NF are composed of natural regenerating trees while PF are composed of planting or seeding trees. In addition, forests whose composition are hard to distinguish or are constituted by mixed trees are also deemed as NF^35^. The forests used in this study thus included 17 NF and 18 PF. Most ecosystems located in the eastern of China, with the northernmost ecosystem appearing in Huzhong cool temperate coniferous forest, and the southernmost ecosystem appearing in Jianfengling tropical seasonal rainforests, covering the major forest types in China. The ecosystems used in this study are shown in Fig. 6, with the detailed information listed in Supplementary Tables S4–S5.

### Data collection

#### Carbon flux data

The carbon flux data used in this study were all from published works. If an ecosystem had multiple references reporting the same year carbon fluxes, we used the reference having the longest carbon flux records. When collecting carbon flux data, we simultaneously collected the geographical information of each ecosystem.

#### Auxiliary data

The auxiliary data used for revealing the differences in NEP between forest types included mean climatic data, seasonal climatic data, soil factors, and biotic factors. Given the measurements of each ecosystem had different record lengths and covered divergent periods, we extracted auxiliary data from their corresponding grid products covering the whole period. Then the mean of the whole period was calculated as the auxiliary data used in this study.

The grid climatic data with a spatial resolution of 30 arc *sec* (~ 1 km) were downscaled from the long time series data of the Climate Research Unit (CRU) by Delta spatial downscaling^47^. The aridity index was also another mean annual climatic factor, which calculated as MAP/PET, where PET was the annual potential evapotranspiration. PET was extracted from the Delta spatial downscaling CRU grid data with a spatial resolution of 30 arc *sec* (~ 1 km)^47^.

Seasonal climatic data included the temperature of the warmest month (T_w_), the temperature of the coldest month (T_c_), the temperature annual range (T_r_), the precipitation of the wettest month (P_w_), the precipitation of the driest month (P_d_), and the precipitation seasonality (P_s_). All seasonal climatic data were extracted from the monthly Delta downscaling long time series data of the Climate Research Unit (CRU)^47^, where Ps was calculated as the standard deviation of monthly precipitation during the measuring year.

Soil factors employed the soil fertilizer variables, including soil organic carbon content (SOC) and soil total nitrogen content (STN), which were extracted from a global soil dataset for use in earth system models (GSDE)^48^.

Biotic factors included leaf area indices and stand age (SA). Leaf area indices were extracted from MODIS products (a spatial resolution of 500 m and a temporal resolution of 8 days)^49^ of each ecosystem. The mean leaf area index (LAI) and maximum leaf area index (MLAI) were calculated based on the extracted data. Stand age (SA) was gathered during the collection of the carbon fluxes. When some ecosystems missed reporting their stand age, we used the descriptions of the forest growth stage to estimate their stand age values by compiling the empirical relationship between stand age and forest growth stage.

### Data analysis

Although we focused on revealing the differences in NEP values and spatial patterns between NF and PF, our understanding of NEP differences would benefit from analyzing the differences in NEP components (GPP and ER). We thus investigated the differences in carbon flux values and spatial patterns. In addition, the differences in auxiliary data between forest types, which benefited the understanding of the differences in carbon fluxes and thus NEP, were also investigated. Before analyzing NEP differences between NF and PF, we corrected carbon fluxes and auxiliary data as those measurements of different ecosystems covered divergent measurement periods.

#### Data corrections

Considering different ecosystems had divergent record lengths and covered different periods, the carbon flux data were corrected with the time series analysis before they were used in further analysis, which avoided the effects of their interannual variations on our main conclusion. In carbon flux corrections, the observed carbon fluxes were corrected with their interannual trends. The carbon fluxes before the measurements were interpolated with the earliest measurements, the interannual trends, and the difference in years, while those after the measurements were interpolated with the latest measurements, the interannual trends, and the difference in years. In this way, the record lengths and the covering periods were consistent among ecosystems, which all ranged from 2003 to 2019. Using the mean values of the whole period as the corrected carbon fluxes, we conducted our analysis focusing on the differences in NEP values and spatial patterns between forest types.

The interannual trends of carbon fluxes were calculated by forest types. Using specific interannual trends for each ecosystem was impossible as most ecosystems having a record length < 5 years. Among 35 ecosystems, we only took 8 ecosystems having ≥ 5 year measurements, including 4 NFs and 4 PFs. The interannual trends of each ecosystem were calculated with the linear regression and the regression slopes were deemed as the interannul trends. The interannual trend of NF was thus calculated as the mean of 4 NFs, so was that of PF. The interannual trends of ecosystems covering longer period (≥ 5 years) and each forest type were listed as Supplementary Table S6. The corrected carbon fluxes were listed as Supplementary Table S4.

Besides the corrected carbon fluxes, we also kept the mean of original measurements.

Given all auxiliary data were extracted from their corresponding grid data, auxiliary data covered the whole period. Then their mean values were calculated. Therefore, the auxiliary data did not need to be corrected.

#### Differences in auxiliary data and carbon flux values

The differences in auxiliary data between forest types were analyzed with ANOVA, which was also employed to reveal the differences in carbon flux values. In addition, the differences in carbon flux values between forest types were analyzed with ANCOVA given the effects of MAT and MAP on carbon flux values, to exclude the potential effects of environmental factors on carbon fluxes. All analysis were conducted both with corrected values and original measurements.

#### Differences in spatial variations of carbon fluxes

The spatial variations of carbon fluxes included their geographical patterns, their responses to environmental factors (climatic, soil, and biological drivers), and their spatial couplings.

Using the geographical information and carbon fluxes of each ecosystem, we analyzed the spatial variations of carbon fluxes along the geographical information and their differences between forest types. Considering the ecosystems used in our study primarily locate in the eastern China and have little variance in their longitude and altitude (Fig. 6), we only analyzed the variations of carbon fluxes with increasing latitude.

With the auxiliary data and corrected carbon fluxes of each ecosystem, we investigated the effects of various factors (climatic, soil, and biological factors) on the spatial variations of carbon fluxes and their differences between forest types with the correlation analysis, which would reveal the effects of main factors on NEP spatial variations.

To further reveal the mechanisms underlying NEP spatial patterns, we analyzed the responses of NEP and its components to environmental factors and their differences between forest types. Given the high correlations among annual climatic factors and seasonal climatic factors (Supplementary Table S2), the partial correlation analysis was firstly conducted to reveal which factors should be employed in investigating their effects on NEP spatial variations. Then the effects of the selected factors on NEP spatial variations and their differences between forest types were investigated with the regression analysis.

The spatial couplings between carbon fluxes and their differences between forest types were investigated with the collected carbon fluxes.

The differences in regression slopes between forest types for each significant spatial variation were tested with ANCOVA.

At the end of the analysis, we constructed linear regressions with “*regstats*” at Matlab considering the category variable (forest type) and environmental factors and their interactions on NEP and its components, to illustrate the differences in NEP spatial patterns. Only variables having significant regression coefficient (p < 0.05) were introduced into the regressions.

#### Uncertainty analysis

Given that we focused on the differences in NEP values and spatial patterns, the mean of corrected carbon fluxes had some uncertainties as the uncertainties in calculating the interannual trends. To illustrate whether uncertainties in carbon flux correction affected our main conclusions, we added a random error to the mean corrected carbon fluxes and recalculated their spatial variations. This process was repeated 100 times. The random errors of NEP values were within 20% of the mean values, while those of GPP and ER were within 10% of the mean values. The statistics of the repeated 100 -time spatial variations were then compared with those calculated from the mean carbon fluxes.

### Statistical analysis

One-way analysis of variance (ANOVA) was employed to analyze the differences in auxiliary data between forest types with an *α* of 0.05, while the differences in carbon fluxes between forest types were analyzed with Analysis of Covariance (ANCOVA) given the effects of MAT and MAP. A correlation analysis was employed to analyze the effects of environmental factors on the spatial variations of carbon fluxes and the interactions among environmental factors. Then a generalized linear regression was used to reveal the effects of MAT on carbon flux spatial variations, to clarify their differences between forest types. The spatial couplings between carbon fluxes were also conducted with generalized linear regression. ANCOVA was employed to reveal the difference in the responses of carbon fluxes to environmental factors and the spatial couplings between carbon fluxes. All analyses were conducted at MATLAB 2014a (MathWorks Inc.).

## Supplementary Information

Supplementary Information.

## Data Availability

The datasets analyzed during the current study are listed in Supplementary Materials. All datasets generated during the current study are available immediately after its publication.
