# Supplementary material for: Eddy covariance-based differences in net ecosystem productivity values and spatial patterns between naturally regenerating forests and planted forests in China
Source: Sci Rep. 2022 Nov 29;12:20556. doi: 10.1038/s41598-022-25025-4 (PMC9709070; doi:10.1038/s41598-022-25025-4)
Supplement: Supplementary file 1 — Supplementary Information. [file 41598_2022_25025_MOESM1_ESM.docx]

Supplementary Information

**Eddy covariance based differences in net ecosystem productivity values and spatial patterns between naturally regenerating forests and planted forests in China**

Xian-Jin Zhu^1,4^, Ren-Xue Fan^1^, Zhi Chen^2,3^, Qiu-Feng Wang^2,3*^, Gui-Rui Yu^2,3*^

1. College of Agronomy, Shenyang Agricultural University, Shenyang 110866, China

2. Synthesis Research Center of Chinese Ecosystem Research Network, Key Laboratory of Ecosystem Network Observation and Modeling, Institute of Geographic Sciences and Natural Resources Research, Chinese Academy of Sciences, Beijing 100101, China;

3. College of Resources and Environment, University of Chinese Academy of Sciences, Beijing 100049, China

4. Liaoning Panjin Wetland Ecosystem National Observation and Research Station, Shenyang, 110866, China

*Corresponding author:

Prof. Qiu-Feng Wang, Prof. Gui-Rui Yu

Institute of Geographic Sciences and Natural Resources Research, Chinese Academy of Sciences

11A Datun Road, Chaoyang District,

Beijing 100101, China

E-Mail: [qfwang@igsnrr.ac.cn](mailto:qfwang@igsnrr.ac.cn), yugr@igsnrr.ac.cn

Tel: 86-10-64889432

Fax: 86-10-64889432

**Supplementary Table S1** Correlation coefficients between carbon fluxes and environmental factors in naturally regenerating forests (NF) and planted forests (PF) with the original measurements

| Environmental factors | NEP | | GPP | | ER | |
| --- | --- | --- | --- | --- | --- | --- |
|  | NF | PF | NF | PF | NF | PF |
| MAT | 0.33 | 0.60 | 0.69 | 0.76 | 0.62 | 0.64 |
| MAP | 0.49 | 0.42 | 0.73 | 0.59 | 0.56 | 0.47 |
| AI | 0.23 | 0.49 | 0.42 | 0.61 | 0.40 | 0.51 |
| Tw | 0.41 | 0.44 | 0.42 | 0.65 | 0.29 | 0.69 |
| Tc | 0.48 | 0.70 | 0.68 | 0.77 | 0.52 | 0.53 |
| Tr | -0.41 | -0.61 | -0.63 | -0.56 | -0.50 | -0.27 |
| Pw | 0.36 | 0.33 | 0.61 | 0.64 | 0.51 | 0.57 |
| Pc | 0.57 | 0.01 | 0.41 | 0.15 | 0.20 | 0.27 |
| Ps | 0.25 | 0.36 | 0.59 | 0.71 | 0.54 | 0.54 |
| LAI | -0.04 | 0.53 | 0.68 | 0.45 | 0.74 | 0.18 |
| MLAI | -0.06 | 0.36 | 0.32 | 0.27 | 0.37 | 0.04 |
| SA | 0.13 | -0.06 | 0.27 | -0.35 | 0.19 | -0.52 |
| SOC | 0.27 | -0.31 | -0.07 | -0.34 | -0.15 | -0.19 |
| STN | 0.23 | -0.37 | -0.14 | -0.26 | -0.20 | -0.05 |

Note: carbon fluxes included net ecosystem productivity (NEP), gross primary productivity (GPP), and ecosystem respiration (ER). The abbreviations of the environmental factors are the same as those in the legend of Figure 1. Grey and dark colors indicate significant correlation coefficients at *α*=0.05 and *α*=0.01, respectively.

**Supplementary Table S2** Correlation coefficients between environmental factors in naturally regenerating forests (NF) and planted forests (PF)

**PF**

**NF**

| **NF** | **Latitude** | **MAT** | **MAP** | **AI** | **Tw** | **Tc** | **Tr** | **Pw** | **Pc** | **Ps** | **LAI** | **MLAI** | **SA** | **SOC** | **STN** |
| --- | --- | --- | --- | --- | --- | --- | --- | --- | --- | --- | --- | --- | --- | --- | --- |
| **Latitude** |  | **-0.73** | **-0.86** | **-0.23** | **-0.35** | **-0.96** | **0.94** | **-0.87** | **-0.49** | **-0.84** | **-0.44** | **-0.06** | **-0.16** | **0.21** | **0.35** |
| **MAT** | **-0.89** |  | **0.66** | **0.58** | **0.78** | **0.82** | **-0.60** | **0.68** | **0.48** | **0.61** | **0.34** | **0.03** | **-0.47** | **-0.47** | **-0.51** |
| **MAP** | **-0.81** | **0.85** |  | **0.05** | **0.46** | **0.87** | **-0.80** | **0.94** | **0.58** | **0.91** | **0.63** | **0.29** | **0.16** | **0.16** | **0.01** |
| **AI** | **-0.88** | **0.84** | **0.63** |  | **0.39** | **0.37** | **-0.25** | **0.09** | **-0.16** | **0.12** | **-0.02** | **-0.23** | **-0.39** | **-0.80** | **-0.73** |
| **Tw** | **-0.57** | **0.80** | **0.72** | **0.56** |  | **0.48** | **-0.13** | **0.46** | **0.62** | **0.34** | **0.33** | **0.21** | **-0.69** | **-0.22** | **-0.19** |
| **Tc** | **-0.98** | **0.93** | **0.82** | **0.89** | **0.68** |  | **-0.93** | **0.88** | **0.46** | **0.86** | **0.44** | **0.05** | **0.07** | **-0.27** | **-0.42** |
| **Tr** | **0.98** | **-0.81** | **-0.71** | **-0.87** | **-0.42** | **-0.95** |  | **-0.80** | **-0.25** | **-0.83** | **-0.36** | **0.03** | **-0.37** | **0.22** | **0.39** |
| **Pw** | **-0.72** | **0.72** | **0.86** | **0.59** | **0.55** | **0.68** | **-0.62** |  | **0.55** | **0.98** | **0.56** | **0.23** | **0.13** | **0.09** | **-0.03** |
| **Pc** | **-0.47** | **0.55** | **0.67** | **0.35** | **0.71** | **0.49** | **-0.31** | **0.48** |  | **0.37** | **0.24** | **0.07** | **-0.29** | **0.12** | **0.10** |
| **Ps** | **-0.71** | **0.68** | **0.79** | **0.59** | **0.43** | **0.66** | **-0.64** | **0.98** | **0.30** |  | **0.58** | **0.25** | **0.24** | **0.11** | **-0.02** |
| **LAI** | **-0.64** | **0.67** | **0.65** | **0.64** | **0.47** | **0.67** | **-0.63** | **0.66** | **0.09** | **0.71** |  | **0.87** | **0.07** | **0.35** | **0.26** |
| **MLAI** | **-0.21** | **0.25** | **0.30** | **0.29** | **0.29** | **0.26** | **-0.20** | **0.33** | **0.01** | **0.34** | **0.75** |  | **0.02** | **0.50** | **0.48** |
| **SA** | **-0.19** | **0.07** | **0.11** | **0.14** | **-0.24** | **0.12** | **-0.24** | **0.20** | **-0.23** | **0.28** | **0.36** | **0.27** |  | **0.35** | **0.19** |
| **SOC** | **-0.13** | **-0.22** | **-0.21** | **0.03** | **-0.34** | **0.06** | **-0.22** | **-0.08** | **-0.16** | **-0.01** | **-0.09** | **0.09** | **0.13** |  | **0.95** |
| **STN** | **-0.16** | **-0.17** | **-0.25** | **0.08** | **-0.31** | **0.09** | **-0.24** | **-0.17** | **-0.16** | **-0.10** | **-0.14** | **0.00** | **0.00** | **0.93** |  |

MAT: mean annual air temperature; MAP: mean annual precipitation; AI: aridity index; LAI: mean annual leaf area index; MLAI: maximum annual leaf area index; NEP: net ecosystem productivity; GPP: gross primary productivity; ER: ecosystem respiration, T_w_: the temperature of warmest month; T_c_: the temperature of coldest month; T_R_: temperature annual range; P_w_: precipitation of wettest month, P_d_: precipitation of driest month, P_S_: precipitation seasonality defined as the standard deviation of monthly precipitation during the measuring year, SA: stand age; SOC: soil organic carbon content, STN: soil total nitrogen content. Grey and dark colors indicated significant correlation coefficients at α=0.05 and α=0.01, respectively.

**Supplementary Table S3** Partial correlation coefficients between carbon fluxes and environmental factors in naturally regenerating forests (NF) and planted forests (PF) with fixing mean annual air temperature (MAT) or mean annual precipitation (MAP) using the original measurements.

| Fixed variable | Environmental factors | NEP | | GPP | | ER | |
| --- | --- | --- | --- | --- | --- | --- | --- |
|  |  | NF | PF | NF | PF | NF | PF |

| MAT | MAP | 0.42 | 0.06 | 0.39 | 0.14 | 0.14 | 0.04 |
| --- | --- | --- | --- | --- | --- | --- | --- |
|  | AI | -0.07 | 0.24 | -0.25 | 0.25 | -0.16 | 0.17 |
|  | Tw | 0.26 | -0.05 | -0.24 | 0.11 | -0.35 | 0.38 |
|  | Tc | 0.48 | 0.47 | 0.16 | 0.42 | -0.11 | 0.03 |
|  | Tr | -0.27 | -0.40 | -0.24 | -0.28 | -0.08 | 0.11 |
|  | Pw | 0.19 | -0.02 | 0.28 | 0.36 | 0.18 | 0.30 |
|  | Pc | 0.50 | -0.21 | 0.15 | -0.11 | -0.11 | 0.11 |
|  | Ps | 0.03 | 0.03 | 0.25 | 0.43 | 0.23 | 0.21 |
|  | LAI | -0.31 | 0.43 | 0.48 | 0.17 | 0.59 | -0.17 |
|  | MLAI | -0.12 | 0.37 | 0.36 | 0.12 | 0.40 | -0.17 |
|  | SA | 0.16 | 0.30 | 0.41 | 0.23 | 0.27 | -0.20 |
|  | SOC | 0.38 | -0.05 | 0.37 | 0.16 | 0.17 | 0.27 |
|  | STN | 0.31 | -0.11 | 0.23 | 0.23 | 0.08 | 0.43 |
| MAP | MAT | -0.18 | 0.46 | 0.26 | 0.59 | 0.33 | 0.49 |
|  | AI | -0.09 | 0.56 | 0.10 | 0.70 | 0.17 | 0.54 |
|  | Tw | 0.11 | 0.31 | -0.12 | 0.54 | -0.13 | 0.61 |
|  | Tc | 0.15 | 0.70 | 0.22 | 0.62 | 0.14 | 0.30 |
|  | Tr | -0.10 | -0.50 | -0.28 | -0.26 | -0.20 | 0.08 |
|  | Pw | -0.16 | -0.13 | -0.02 | 0.33 | 0.07 | 0.36 |
|  | Pc | 0.37 | -0.22 | -0.14 | -0.33 | -0.28 | -0.02 |
|  | Ps | -0.31 | 0.00 | 0.04 | 0.47 | 0.19 | 0.30 |
|  | LAI | -0.56 | 0.41 | 0.40 | 0.19 | 0.59 | -0.10 |
|  | MLAI | -0.30 | 0.30 | 0.12 | 0.10 | 0.23 | -0.13 |
|  | SA | 0.17 | -0.14 | 0.39 | -0.33 | 0.22 | -0.51 |
|  | SOC | 0.37 | -0.43 | 0.19 | -0.34 | -0.01 | -0.16 |
|  | STN | 0.35 | -0.41 | 0.20 | -0.20 | 0.01 | 0.03 |

Note: carbon fluxes included net ecosystem productivity (NEP), gross primary productivity (GPP), and ecosystem respiration (ER). The abbreviations of the environmental factors are the same as those in the legend of Figure 1. Grey and dark colors indicate significant correlation coefficients at *α*=0.05 and *α*=0.01, respectively.

**Supplementary Table S4** Brief information of sites used in this study

| ID | Name | Latitude (°N) | Longitude(°E) | LAI (m^2^ m^-2^) | MLAI  (m^2^ m^-2^) | Corrected values (gC m^-2^ yr^-1^) | | | Original measurements (gC m^-2^ yr^-1^) | | | Measuring period | Forest type^2^ | Reference |
| --- | --- | --- | --- | --- | --- | --- | --- | --- | --- | --- | --- | --- | --- | --- |
|  |  |  |  |  |  | NEP | GPP | ER | NEP | GPP | ER |  |  |  |
| 1 | Jianfengling | 18.73 | 108.8 | 4.55 | 6.32 | 174.68 | 1916.93 | 1741.99 | 235.50 | 1970.00 | 1734.50 | 2006-2009 | N | [^1^](#_ENREF_1) |
| 2 | Danzhou | 19.55 | 109.48 | 2.93 | 4.43 | 1125.63 | 3143.17 | 2017.65 | 1133.45 | 3143.93 | 2010.48 | 2010 | P | [^2^](#_ENREF_2) |
| 3 | Wenchang | 19.72 | 110.95 | 1.40 | 2.25 | 440.06 | - | - | 400.96 | - | - | 2016 | P | [^3^](#_ENREF_3) |
| 4 | Xishuangbanna rubber | 21.93 | 101.27 | 2.38 | 3.38 | 904.00 | 1816.00 | 913.00 | 904.00 | 1816.00 | 913.00 | 2011 | P | [^4^](#_ENREF_4) |
| 5 | Xishuangbanna | 21.95 | 101.2 | 5.03 | 6.39 | 111.18 | 2333.34 | 2221.91 | 127.31 | 2445.63 | 2318.31 | 2003-2010 | N | [^5^](#_ENREF_5) |
| 6 | Dinghushan | 23.17 | 112.54 | 3.58 | 5.54 | 338.27 | 1413.75 | 1075.25 | 413.20 | 1413.89 | 1000.69 | 2003-2010 | N | [^6^](#_ENREF_6)^,^[^7^](#_ENREF_7) |
| 7 | Xitou | 23.67 | 120.80 | 3.64 | 5.59 | 623.84 | - | - | 561.33 | - | - | 2011-2013 | P | [^8^](#_ENREF_8) |
| 8 | Ailaoshan | 24.54 | 101.03 | 3.27 | 5.14 | 698.92 | 1866.68 | 1055.57 | 542.00 | 1678.50 | 1086.00 | 2009-2016 | N | [^9^](#_ENREF_9) |
| 9 | Qianyanzhou | 26.74 | 115.061 | 3.70 | 6.02 | 468.49 | 1727.47 | 1259.08 | 461.80 | 1723.97 | 1262.16 | 2003-2014 | P | [^10^](#_ENREF_10) |
| 10 | Huitong | 26.83 | 109.75 | 1.86 | 3.75 | 231.84 | 1211.32 | 979.81 | 255.30 | 1213.60 | 958.30 | 2008 | P | [^11^](#_ENREF_11) |
| 11 | Lijiang | 27.14 | 100.23 | 1.15 | 2.54 | 447.21 | 1397.46 | 950.52 | 405.00 | 1392.00 | 987.00 | 2014 | N | [^12^](#_ENREF_12) |
| 12 | Ningxiang | 28.33 | 112.57 | 1.68 | 3.44 | 456.94 | 1967.24 | 1510.48 | 428.80 | 1963.60 | 1534.80 | 2013 | N | [^13^](#_ENREF_13) |
| 13 | Yueyang | 29.53 | 112.86 | 1.26 | 3.80 | 409.88 | 1918.97 | 1509.77 | 466.72 | 1864.07 | 1397.74 | 2006-2013 | P | [^14^](#_ENREF_14) |
| 14 | Gonggashan | 29.57 | 101.985 | 0.67 | 1.85 | 273.15 | 809.72 | 536.13 | 241.87 | 806.68 | 564.81 | 2015 | P | [^15^](#_ENREF_15) |
| 15 | Jinyunshan | 29.78 | 106.34 | 2.56 | 5.10 | 636.84 | 1770.73 | 1135.88 | 566.49 | 1761.63 | 1196.68 | 2016 | N | [^16^](#_ENREF_16) |
| 16 | Linan | 30.18 | 119.34 | 1.36 | 2.42 | 115.64 | 1341.36 | 1225.53 | 104.22 | 1404.40 | 1300.00 | 2011-2016 | P | [^17^](#_ENREF_17) |
| 17 | Tianmushan | 30.35 | 119.44 | 1.93 | 5.18 | 775.58 | 1661.57 | 886.25 | 737.11 | 1655.29 | 918.19 | 2013-2016 | N | [^18^](#_ENREF_18) |
| 18 | Anqing | 30.47 | 116.99 | 0.83 | 1.76 | 520.79 | 1896.64 | 1376.32 | 506.10 | 1859.20 | 1353.10 | 2006-2007 | P | [^19^](#_ENREF_19) |
| 19 | Anji | 30.48 | 119.67 | 2.55 | 4.93 | 630.09 | 1553.59 | 921.34 | 600.28 | 1540.00 | 938.40 | 2011-2016 | P | [^20^](#_ENREF_20) |
| 20 | Fengxian | 30.84 | 121.51 | 0.83 | 1.70 | 531.28 | - | - | 500.00 | - | - | 2015 | P | [^21^](#_ENREF_21) |
| 21 | Jurong | 32.12 | 119.2 | 1.87 | 4.06 | 133.84 | 1113.00 | 1031.46 | 152.03 | 1118.46 | 994.98 | 2008-2011 | N | [^22^](#_ENREF_22) |
| 22 | Xiping | 33.35 | 113.91 | 1.48 | 3.98 | 335.58 | 1287.34 | 971.87 | 343.40 | 1288.10 | 964.70 | 2010 | P | [^23^](#_ENREF_23) |
| 23 | Baotianman | 33.50 | 111.94 | 2.35 | 5.41 | 653.82 | 1110.22 | 456.94 | 569.40 | 1099.30 | 529.90 | 2010-2013 | N | [^24^](#_ENREF_24) |
| 24 | Qinling | 33.46 | 108.48 | 2.01 | 5.20 | 303.21 | 1081.83 | 778.65 | 288.33 | 1068.70 | 780.37 | 2017 | N | [^25^](#_ENREF_25) |
| 25 | Xiaolangdi | 35.02 | 112.47 | 1.60 | 3.92 | 436.68 | 1241.49 | 805.28 | 497.25 | 1235.94 | 738.69 | 2006-2007 | P | [^26^](#_ENREF_26) |
| 26 | Daxing | 39.53 | 116.25 | 0.73 | 2.20 | 774.58 | 1521.20 | 1098.85 | 677.33 | 1525.00 | 1063.00 | 2006-2008 | P | [^27^](#_ENREF_27) |
| 27 | Aolinpike park | 40.02 | 116.38 | 0.98 | 2.64 | 210.46 | 1255.31 | 1008.88 | 203.80 | 1245.50 | 1002.50 | 2012-2013 | P | [^28^](#_ENREF_28) |
| 28 | Badaling | 40.37 | 115.94 | 0.56 | 1.56 | -221.61 | 699.65 | 921.21 | -226.50 | 699.00 | 925.50 | 2011-2012 | P | [^29^](#_ENREF_29) |
| 29 | Songshan | 40.51 | 115.79 | 1.52 | 4.24 | 223.56 | 680.56 | 457.72 | 111.00 | 666.00 | 555.00 | 2019 | N | [^30^](#_ENREF_30) |
| 30 | Changbaishan | 42.40 | 128.10 | 2.29 | 6.11 | 115.76 | 1388.49 | 1272.49 | 241.01 | 1463.37 | 1222.36 | 2003-2010 | N | [^31^](#_ENREF_31) |
| 31 | Laoshan | 45.33 | 127.67 | 2.42 | 6.42 | 196.15 | 1086.98 | 891.88 | 204.66 | 1166.24 | 962.58 | 2004-2008 | P | [^32^](#_ENREF_32)^,^[^33^](#_ENREF_33) |
| 32 | Maoershan | 45.42 | 127.67 | 2.46 | 6.41 | 164.11 | 1305.95 | 1144.19 | 160.50 | 1341.08 | 1181.25 | 2005-2018 | N | [^34^](#_ENREF_34) |
| 33 | Yichun Old | 48.1 | 129.23 | 2.25 | 5.01 | 132.89 | - | - | 199.00 | - | - | 2007-2008 | N | [^35^](#_ENREF_35) |
| 34 | Yichun Young | 48.12 | 129.25 | 0.24 | 0.71 | 170.11 | - | - | 212.32 | - | - | 2008 | N | [^35^](#_ENREF_35) |
| 35 | Huzhong | 51.78 | 123.02 | 1.64 | 5.36 | 6.50 | 718.44 | 586.90 | 54.28 | 739.03 | 563.30 | 2007-2008 | N | [^36^](#_ENREF_36) |

Note: 1 LAI: mean annual leaf area index; MLAI: maximum annual leaf area index; NEP: net ecosystem productivity; GPP: gross primary productivity; ER: ecosystem respiration

2 N: naturally regenerating forests; P: planted forests

**Supplementary Table S5** Auxiliary data of sites used in this study

| ID | Name | Latitude (°N) | Longitude(°E) | MAT^1^ (℃) | MAP (mm) | AI | T_W_ (℃) | T_c_ (℃) | T_R_ (℃) | P_W_ (mm) | P_d_ (mm) | P_S_ (mm) | SA (year) | SOC (gC m^-2^) | STN (gN m^-2^) | Leaf type^2^ |
| --- | --- | --- | --- | --- | --- | --- | --- | --- | --- | --- | --- | --- | --- | --- | --- | --- |
| 1 | Jianfengling | 18.73 | 108.8 | 20.78 | 1614.36 | 1.30 | 29.14 | 20.04 | 9.09 | 449.34 | 17.03 | 143.13 | 26.50 | 1841.67 | 133.38 | E |
| 2 | Danzhou | 19.55 | 109.48 | 24.43 | 1706.50 | 1.41 | 29.52 | 18.46 | 11.06 | 443.74 | 18.25 | 144.18 | 9 | 326.25 | 50.63 | E |
| 3 | Wenchang | 19.72 | 110.95 | 24.65 | 1801.38 | 1.75 | 28.11 | 17.57 | 10.54 | 460.39 | 16.68 | 143.51 | 35 | 410.63 | 28.13 | E |
| 4 | Xishuangbanna rubber | 21.93 | 101.27 | 23.31 | 1661.70 | 1.47 | 25.29 | 16.95 | 8.35 | 288.09 | 4.51 | 96.10 | 33 | 360 | 22.50 | E |
| 5 | Xishuangbanna | 21.95 | 101.2 | 22.46 | 1623.37 | 1.44 | 25.29 | 16.95 | 8.35 | 288.09 | 4.51 | 96.10 | 200 | 360 | 22.50 | E |
| 6 | Dinghushan | 23.17 | 112.54 | 21.11 | 1806.77 | 1.73 | 27.92 | 12.97 | 14.95 | 453.71 | 10.28 | 143.92 | 216.67 | 820.80 | 64.80 | E |
| 7 | Xitou | 23.67 | 120.80 | 17.40 | 2367.04 | 2.55 | 28.69 | 17.75 | 10.95 | 491.48 | 20.13 | 159.59 | 69 | 2371.95 | 146.48 | E |
| 8 | Ailaoshan | 24.54 | 101.03 | 12.56 | 916.52 | 0.85 | 22.59 | 11.17 | 11.42 | 243.92 | 4.58 | 84.45 | 300 | 2994.66 | 194.54 | E |
| 9 | Qianyanzhou | 26.74 | 115.061 | 18.82 | 1446.06 | 1.37 | 29.59 | 7.22 | 22.36 | 284.71 | 18.42 | 82.02 | 20 | 810.81 | 57.92 | E |
| 10 | Huitong | 26.83 | 109.75 | 17.02 | 1218.36 | 1.44 | 26.30 | 5.18 | 21.12 | 290.35 | 17.85 | 84.53 | 13 | 1386.00 | 89.10 | E |
| 11 | Lijiang | 27.14 | 100.23 | 6.70 | 778.51 | 0.83 | 13.26 | 0.50 | 12.76 | 196.58 | 2.52 | 71.59 | 160 | 3369.60 | 290.16 | E |
| 12 | Ningxiang | 28.33 | 112.57 | 18.15 | 1292.32 | 1.37 | 29.11 | 4.84 | 24.28 | 252.62 | 25.74 | 69.40 | 40 | 853.20 | 86.40 | E |
| 13 | Yueyang | 29.53 | 112.86 | 17.94 | 1201.45 | 1.22 | 29.92 | 5.13 | 24.79 | 242.02 | 22.18 | 65.00 | 5.67 | 969.03 | 84.92 | E |
| 14 | Gonggashan | 29.571 | 101.985 | 1.00 | 846.01 | 0.97 | 15.36 | -0.12 | 15.48 | 196.20 | 0.59 | 68.87 | 120 | 1145.75 | 71.96 | E |
| 15 | Jinyunshan | 29.78 | 106.34 | 17.04 | 1169.08 | 1.35 | 28.78 | 8.31 | 20.46 | 228.81 | 11.29 | 73.30 | 50 | 2081.52 | 166.32 | E |
| 16 | Linan | 30.18 | 119.34 | 16.66 | 1357.95 | 1.46 | 27.09 | 3.05 | 24.04 | 289.43 | 30.61 | 76.27 | 12.14 | 926.33 | 87.98 | E |
| 17 | Tianmushan | 30.35 | 119.44 | 11.30 | 1611.52 | 1.72 | 27.09 | 3.05 | 24.04 | 289.43 | 30.61 | 76.27 | 140 | 1717.20 | 91.80 | E |
| 18 | Anqing | 30.47 | 116.99 | 17.55 | 1468.61 | 1.38 | 29.58 | 4.72 | 24.86 | 278.46 | 24.55 | 78.12 | 17.50 | 976.50 | 83.70 | E |
| 19 | Anji | 30.48 | 119.67 | 15.77 | 1316.50 | 1.38 | 27.86 | 3.55 | 24.31 | 273.37 | 30.45 | 71.38 | 10 | 1074.15 | 79.20 | E |
| 20 | Fengxian | 30.84 | 121.51 | 16.95 | 982.91 | 1.01 | 29.24 | 5.19 | 24.05 | 253.19 | 30.58 | 66.43 | 10 | 802.58 | 71.96 | E |
| 21 | Jurong | 32.12 | 119.2 | 15.63 | 1085.28 | 1.09 | 28.94 | 3.14 | 25.80 | 251.36 | 19.26 | 70.14 | 59 | 1235.25 | 104.31 | E |
| 22 | Xiping | 33.35 | 113.91 | 15.89 | 841.38 | 0.81 | 28.10 | 2.42 | 25.68 | 203.94 | 6.06 | 62.49 | 8 | 442.13 | 53.06 | D |
| 23 | Baotianman | 33.50 | 111.94 | 11.20 | 885.50 | 0.89 | 27.80 | 2.98 | 24.82 | 185.78 | 5.50 | 56.99 | 65 | 1017.45 | 101.75 | D |
| 24 | Qinling | 33.46 | 108.48 | 5.74 | 908.32 | 1.04 | 20.58 | -0.84 | 21.42 | 200.67 | 2.94 | 64.48 | 50 | 2942.55 | 181.35 | D |
| 25 | Xiaolangdi | 35.02 | 112.47 | 14.76 | 604.75 | 0.55 | 26.11 | -0.47 | 26.58 | 170.59 | 1.89 | 53.31 | 30 | 370.62 | 38.34 | D |
| 26 | Daxing | 39.53 | 116.25 | 13.04 | 577.15 | 0.53 | 27.62 | -2.71 | 30.34 | 154.20 | 0.39 | 49.11 | 8.33 | 250.92 | 18.36 | D |
| 27 | Aolinpike park | 40.02 | 116.38 | 13.21 | 526.37 | 0.48 | 24.60 | -6.22 | 30.82 | 140.86 | 0.51 | 45.10 | 20 | 357.12 | 40.32 | D |
| 28 | Badaling | 40.37 | 115.94 | 10.29 | 450.30 | 0.42 | 22.99 | -8.74 | 31.72 | 131.31 | 0.46 | 42.49 | 4 | 992.66 | 98.69 | D |
| 29 | Songshan | 40.51 | 115.79 | 8.78 | 438.01 | 0.41 | 20.83 | -10.92 | 31.75 | 123.99 | 0.55 | 40.44 | 95 | 944.64 | 92.16 | D |
| 30 | Changbaishan | 42.40 | 128.10 | 3.88 | 710.59 | 0.91 | 18.16 | -18.94 | 37.10 | 279.81 | 1.67 | 89.54 | 200 | 2438.28 | 126.36 | D |
| 31 | Laoshan | 45.33 | 127.67 | 3.00 | 629.59 | 0.81 | 22.59 | -17.54 | 40.12 | 154.13 | 2.55 | 51.95 | 37 | 2753.10 | 227.70 | D |
| 32 | Maoershan | 45.42 | 127.67 | 3.00 | 623.01 | 0.80 | 22.59 | -17.54 | 40.12 | 154.13 | 2.55 | 51.95 | 70 | 2753.10 | 227.70 | D |
| 33 | Yichun Old | 48.1 | 129.23 | 1.56 | 656.81 | 1.03 | 19.49 | -23.39 | 42.88 | 180.45 | 2.01 | 60.19 | 220 | 814.05 | 48.24 | D |
| 34 | Yichun Young | 48.117 | 129.25 | 1.48 | 652.21 | 1.02 | 19.49 | -23.39 | 42.88 | 180.45 | 2.01 | 60.19 | 22 | 814.05 | 48.24 | D |
| 35 | Huzhong | 51.78 | 123.02 | -2.93 | 490.55 | 0.83 | 16.24 | -28.30 | 44.54 | 135.96 | 1.93 | 45.43 | 70 | 971.73 | 82.35 | D |

Note: 1 MAT: mean annual air temperature; MAP: mean annual precipitation; AI: aridity index; T_w_: the temperature of warmest month; T_c_: the temperature of coldest month; T_R_: temperature annual range; P_w_: precipitation of wettest month, P_d_: precipitation of driest month, P_S_: precipitation seasonality defined as the standard deviation of monthly precipitation during the measuring year, SA: stand age; SOC: soil organic carbon content, STN: soil total nitrogen content

2 E: evergreen; D: Deciduous

**Supplementary Table S6** The interannual trends of carbon fluxes in ecosystems having longer period measurements (≥ 5 years) and each forest type

| Forest type | Forest site name | Latitude (°N) | Longitude(°E) | Interannual trend (gC m^-2^ yr^-2^) | | |
| --- | --- | --- | --- | --- | --- | --- |
|  |  |  |  | NEP | GPP | ER |
| Naturally regenerating forests (NF) | Xishuangbanna | 21.95 | 101.2 | 2.61 | -49.41 | -52.02 |
|  | Dinghushan | 23.17 | 112.54 | -29.21 | -1.09 | 28.12 |
|  | Changbaishan | 42.40 | 128.10 | -26.17 | 15.06 | 41.24 |
|  | Maoershan | 45.42 | 127.67 | -3.50 | 28.17 | 31.30 |
|  | Mean |  | | -14.07 | -1.82 | 12.16 |
| Planted forests (PF) | Qianyanzhou | 26.74 | 115.061 | 3.99 | 4.58 | 0.59 |
|  | Yueyang | 29.53 | 112.86 | -44.12 | -46.43 | -2.17 |
|  | Linan | 30.18 | 119.34 | 3.01 | 28.50 | 25.35 |
|  | Anqing | 30.47 | 116.99 | 5.85 | 10.29 | 4.92 |
|  | Mean |  | | -7.82 | -0.76 | 7.17 |

**Supplementary Text S1** The equations describing the spatial variations of net ecosystem productivity and its related factors with the original measurements

MAT=35.08-0.68Latitude, R^2^=0.67, n=35, p<0.01 (1)

GPP=770.95+53.25MAT, R^2^=0.48, n=30, p<0.01 (2)

ER=312.29-518.84Forest+0.52GPP+0.39Forest×GPP, R^2^=0.79, n=30, p<0.01 (3)

NEP=-302.66+470.15Forest+0.48GPP-0.37Forest×GPP, R^2^=0.49, n=30, p<0.01 (4)

**References**

1 Chen, D. *Dynamics and controls of carbon exchange of a tropical montane rain forest at Jianfengling, China* Master thesis, Chinese Academy of Forestry, (2010).

2 Wu, Z., Xie, G., Yang, C., Chen, B. & Zhou, Z. Characteristics of carbon fluxes in a rubber plantation ecosystem in Danzhou area, Hainan Province. *Journal of Northwest Forestry University* **30**, 51-59.(In Chinese with English Abstract) (2015).

3 Li, D., Fu, G., Xue, Y. & Yang, Z. Preliminary Analysis of Dynamic Patterns and Causes of Carbon Fluxes in A Tropical Coastal Sandy Vegetation Ecosystem in Hainan. *Natural science journal of Hainan University* **36**, 173-182.(In Chinese with English Abstract) (2018).

4 Song, Q.-H. *et al.* Do the rubber plantations in tropical China act as large carbon sinks? *Iforest-Biogeosciences and Forestry* **7**, 42-47, doi:10.3832/ifor0891-007 (2013).

5 Zhang, Y. P., Tan, Z. H., Song, Q. H., Yu, G. R. & Sun, X. M. Respiration controls the unexpected seasonal pattern of carbon flux in an Asian tropical rain forest. *Atmos. Environ.* **44**, 3886-3893, doi:10.1016/j.atmosenv.2010.07.027 (2010).

6 Yan, J. *et al.* Substantial amounts of carbon are sequestered during dry periods in an old-growth subtropical forest in South China. *Journal of Forest Research* **18**, 21-30, doi:10.1007/s10310-012-0363-0 (2013).

7 Yan, J. *et al.* Seasonal and inter-annual variations in net ecosystem exchange of two old-growth forests in southern China. *Agric. For. Meteorol.* **182–183**, 257-265, doi:<http://dx.doi.org/10.1016/j.agrformet.2013.03.002> (2013).

8 Maneke-Fiegenbaum, F., Klemm, O., Lai, Y.-J., Hung, C.-Y. & Yu, J.-C. Carbon Exchange between the Atmosphere and a Subtropical Evergreen Mountain Forest in Taiwan. *Advances in Meteorology*, doi:10.1155/2018/9287249 (2018).

9 Song, Q.-H. *et al.* Water use efficiency in a primary subtropical evergreen forest in Southwest China. *Sci. Rep.* **7**, 43031, doi:10.1038/srep43031

https://[www.nature.com/articles/srep43031#supplementary-information](http://www.nature.com/articles/srep43031#supplementary-information) (2017).

10 Xu, M. J. *et al.* The full annual carbon balance of a subtropical coniferous plantation is highly sensitive to autumn precipitation. *Sci. Rep.* **7**, doi:10.1038/s41598-017-10485-w (2017).

11 Zhao, Z. *A Study on Carbon Flux between Chinese Fir Planations and Atmosphere in Subtropical Belts* Doctor thesis, Central South University of Forestry and Technology, (2011).

12 Fei, X. *et al.* Carbon exchanges and their responses to temperature and precipitation in forest ecosystems in Yunnan, Southwest China. *Sci. Total Environ.* **616**, 824-840, doi:10.1016/j.scitotenv.2017.10.239 (2018).

13 Jia, B. *et al.* Diurnal and Seasonal Variations of CO2 Fluxes and Their Climate Controlling Factors for a Subtropical Forest in Ningxiang. *Adv. Atmos. Sci.* **32**, 553-564, doi:10.1007/s00376-014-4069-4 (2015).

14 Gao, S. *et al.* Ecosystem carbon (CO2 and CH4) fluxes of a Populus dettoides plantation in subtropical China during and post clear-cutting. *For. Ecol. Manage.* **357**, 206-219, doi:10.1016/j.foreco.2015.08.026 (2015).

15 Zhang, Y., Zhu, W., Sun, X. & Hu, Z. Carbon dioxide flux characteristics in an Abies fabri mature forest on Gongga Mountain, Sichuan, China. *Acta Ecologica Sinica* **38**, 6125-6135.(In Chinese with English Abstract) (2018).

16 Wang, Q., Wang, Y.-q., Ma, C., Wang, B. & Li, Y.-f. Characteristics of Carbon Fluxes and Their Response to Environmental Factors in Ecosystems of Mixed Coniferous and Broad-leaved Forests in Jinyun Mountain. *Resources and Environment in the Yangtze Basin* **28**, 565-576.（In Chinese with English Abstract) (2019).

17 Chen, Y., Jiang, H., Zhou, G., Yang, S. & Chen, J. Estimation of CO_2_ fluxes and its seasonal variations from the effective management Lei bamboo (*Phyllostachys Violascens*). *Acta Ecologica Sinica* **33**, 3434-3444(in Chinese with English abstract), doi:10.5846/stxb201203300444 (2013).

18 Niu, X.-d. *et al.* Characteristics of CO₂ flux in an old growth mixed forest in Tianmu Mountain, Zhejiang, China. *Ying yong sheng tai xue bao = The journal of applied ecology* **27**, 1-8 (2016).

19 Han, S. *Productivity estimation of the poplar plantations on the beaches in middle and low reaches of Yangtze river using eddy covariance measurement* Master thesis, Chinese Academy of Forestry, (2008).

20 Song, X., Chen, X., Zhou, G., Jiang, H. & Peng, C. Observed high and persistent carbon uptake by Moso bamboo forests and its response to environmental drivers. *Agric. For. Meteorol.* **247**, 467-475, doi:10.1016/j.agrformet.2017.09.001 (2017).

21 Zhang, K. *et al.* Measuring Multi-Scale Urban Forest Carbon Flux Dynamics Using an Integrated Eddy Covariance Technique. *Sustainability* **11**, doi:10.3390/su11164335 (2019).

22 Jiang, Y. *The research of North subtropical secondary oak forest carbon budget* Master thesis, Nanjing forestry university, (2010).

23 Geng, S. *Study on the carbon flux observation over poplar plantation ecosystem of XiPing city in Henan Province of China* Master thesis, Beijing Forestry University, (2011).

24 Niu, X., Sun, P., Liu, X., Luan, J. & Liu, S. Net Ecosystem carbon dioxide change in an oak (Quercusaliena) forest at transitional zone from subtropics to warm temperate, China. *Acta Ecologica Sinica* **40**, 5980-5991 (2020).

25 Yuan, J. *et al.* Biometric and Eddy Covariance Methods for Examining the Carbon Balance of a Larix principis-rupprechtii Forest in the Qinling Mountains, China. *Forests* **9**, doi:10.3390/f9020067 (2018).

26 Tong, X., Zhang, J., Meng, P., Li, J. & Zheng, N. Ecosystem water use efficiency in a warm-temperate mixed plantation in the North China. *J. Hydrol.* **512**, 221-228, doi:<http://dx.doi.org/10.1016/j.jhydrol.2014.02.042> (2014).

27 Zha, T. G. *Carbon balance of a poplar plantation ecosystem in Daxing, Beijing* Doctor thesis, Beijing Forestry University, (2007).

28 Xie, J. *et al.* Seasonal variation in ecosystem water use efficiency in an urban-forest reserve affected by periodic drought. *Agric. For. Meteorol.* **221**, 142-151, doi:10.1016/j.agrformet.2016.02.013 (2016).

29 Ma, J. *et al.* Ecosystem water use efficiency in a young plantation in Northern China and its relationship to drought. *Agric. For. Meteorol.* **275**, 1-10, doi:10.1016/j.agrformet.2019.05.004 (2019).

30 Li, R. *et al.* Net ecosystem carbon exchange and its affecting factors in a deciduous broad-leaved forest in Songshan, Beijing, China. *Chinese Journal of Applied Ecology* **31**, 3621-3630 (2020).

31 Yu, G.-R. *et al.* Spatial patterns and climate drivers of carbon fluxes in terrestrial ecosystems of China. *Global Change Biol.* **19**, 798-810, doi:10.1111/gcb.12079 (2013).

32 Wang, H.-m. *et al.* Carbon fluxes and their response to environmental variables in a Dahurian larch forest ecosystem in northeast China. *Journal of Forestry Research* **19**, 1-10, doi:10.1007/s11676-008-0001-z (2008).

33 Qiu, L. *et al.* CO2 flux characteristics and their influence on the carbon budget of a larch plantation in Maoershan region of Northeast China. *Yingyong Shengtai Xuebao* **22**, 1-8 (2011).

34 Wang, X. C. *Temporal variations and environmental control of carbon dioxide exchange of a natural secondary forest in Northeastern China* Master thesis, Northeast Forestry University, (2008).

35 Wang, Y., Zhou, G. S., Jia, B. R., Li, S. & Wang, S. H. Comparisons of carbon flux and its controls between broad-leaved Korean pine forest and Dahurian larch forest in northeast China. *Acta Ecologica Sinica (in Chinese with English abstract)* **30**, 4376-4388 (2010).

36 Zhou, L., Jia, B., Zhou, G., Zeng, W. & Wang, Y. Carbon exchange of Chinese boreal forest during its growth season and related regulation mechanisms. *Chinese Journal of Applied Ecology* **21**, 2449-2456.(in Chinese with English Abstract) (2010).
